# Colocalization of corneal resistance factor GWAS loci with GTEx e/sQTLs highlights plausible candidate causal genes for keratoconus postnatal corneal stroma weakening

**DOI:** 10.3389/fgene.2023.1171217

**Published:** 2023-08-09

**Authors:** Xinyi Jiang, Thibaud Boutin, Veronique Vitart

**Affiliations:** ^1^ MRC Human Genetics Unit, Institute of Genetics and Cancer, University of Edinburgh, Edinburgh, United Kingdom; ^2^ Centre for Genetics and Molecular Medicine, Institute of Genetics and Cancer, University of Edinburgh, Edinburgh, United Kingdom

**Keywords:** cornea, genome-wide association studies, fine-mapping, colocalization, keratoconus, extracellular matrix, genotype–tissue expression, biomechanics min. 5–max. 8

## Abstract

**Background:** Genome-wide association studies (GWAS) for corneal resistance factor (CRF) have identified 100s of loci and proved useful to uncover genetic determinants for keratoconus, a corneal ectasia of early-adulthood onset and common indication of corneal transplantation. In the current absence of studies to probe the impact of candidate causal variants in the cornea, we aimed to fill some of this knowledge gap by leveraging tissue-shared genetic effects.

**Methods:** 181 CRF signals were examined for evidence of colocalization with genetic signals affecting steady-state gene transcription and splicing in adult, non-eye, tissues of the Genotype-Tissue Expression (GTEx) project. Expression of candidate causal genes thus nominated was evaluated in single cell transcriptomes from adult cornea, limbus and conjunctiva. Fine-mapping and colocalization of CRF and keratoconus GWAS signals was also deployed to support their sharing causal variants.

**Results and discussion:** 26.5% of CRF causal signals colocalized with GTEx v8 signals and nominated genes enriched in genes with high and specific expression in corneal stromal cells amongst tissues examined. Enrichment analyses carried out with nearest genes to all 181 CRF GWAS signals indicated that stromal cells of the limbus could be susceptible to signals that did not colocalize with GTEx’s. These cells might not be well represented in GTEx and/or the genetic associations might have context specific effects. The causal signals shared with GTEx provide new insights into mediation of CRF genetic effects, including modulation of splicing events. Functionally relevant roles for several implicated genes’ products in providing tensile strength, mechano-sensing and signaling make the corresponding genes and regulatory variants prime candidates to be validated and their roles and effects across tissues elucidated. Colocalization of CRF and keratoconus GWAS signals strengthened support for shared causal variants but also highlighted many ways into which likely true shared signals could be missed when using readily available GWAS summary statistics.

## 1 Introduction

The cornea requires specific biomechanical and physical properties to enable a dome-like, transparent, protective, and highly refractive structure necessary for clear vision. Genome-wide association studies (GWASs) have given support to the notion that many genetic determinants of inter-individual variability in quantitative measures of those properties also contribute to disease risk. Identifying the causal variants for these associations and how they exert their effect could thus provide valuable pathogenic insights.

To date, GWAS for central corneal thickness (CCT) and corneal resistance factor (CRF) have proved particularly useful to inform on keratoconus susceptibility. Keratoconus is characterized by postnatal progressive thinning and weakening within the central cornea, manifesting by the surface of the eye adopting an irregular and distorted shape with localized steepening. Alterations in the collagen fibrillar structure constitutive of the corneal stroma underpin these changes (Meek et al., 2005), resulting in visual impairments, from myopia, irregular astigmatism and, in advanced cases, tissue scarring. Transcriptomics and proteomics have provided clues on molecular dysfunctions both in the stromal and epithelial layers of the cornea (Yam et al., 2019), but how they arise remains poorly understood (Davidson et al., 2014). Up to 16 keratoconus risk loci were first identified by testing the effects of variants yielded by CCT GWAS (Lu et al., 2013; Cuellar-Partida et al., 2015; Iglesias et al., 2018; Choquet et al., 2020) in, small, keratoconus case–control cohorts; those variants also associate with CRF (Jiang et al., 2020; Simcoe et al., 2020). A recent multi-ancestry keratoconus GWAS meta-analysis has yielded 36 loci reaching genome-wide significance (Hardcastle et al., 2021), 20 of which overlap with CRF or CCT loci known at the time or since established (He et al., 2022). Leveraging the suspected large contribution of CRF/CCT causal variants to disease risk, additional candidate risk loci (18 novel) have been subsequently extracted from the keratoconus GWAS results not reaching genome-wide significance (He et al., 2022).

Pleiotropy of genetic associations allows to propagate functional insights. Evidencing that causal signals for a trait of interest also underpin mRNA- or protein-level modulations is particularly useful, informing on both causal variants’ function and the gene products plausibly mediating impact on trait (Albert and Kruglyak, 2015). With the current lack of GWAS for transcript or protein levels in the directly relevant corneal cells or tissues, we aim to exploit here the notion that a fraction of regulatory variants acts in the same or similar molecular way across multiple tissues in the body, so that effects in corneal cells could be extrapolated from those exerted in non-corneal tissues. Cross-tissues sharing has been extensively studied for the well-characterized catalog of genetic variation affecting steady-state transcript levels in 49 adult, non-ocular, tissues or cells from the Genotype–Tissue Expression (GTEx) consortium (Aguet et al., 2020). That of expression (e)QTLs was shown to be greater for those acting in cis than those acting in trans, and sharing distribution appears U-shaped, with cis-eQTLs discovered in only a few or many tissues (Aguet et al., 2017; Aguet et al., 2020). Regulatory effects sharing has been shown to increase with tissues’ similarity, as evaluated from the patterns of gene expression or, inferred, major cell types' composition (Aguet et al., 2020), consistent with sharing of regulatory features across biologically related cell types (Meuleman et al., 2020). We previously reported significant enrichments of CRF GWAS variants in regions bearing hallmarks of regulatory regions in a wide range of tissues and cells, such as in the lungs, heart, skin, and fibroblasts (Jiang et al., 2020). This supports that CRF causal variants located in these regions might also underpin molecular quantitative trait loci (QTLs) detected in GTEx projects. Splicing (s)QTLs could be particularly informative as exerting similar effects across tissues expressing implicated isoforms (Aguet et al., 2020), while shared cis-eQTLs would require cautious interpretation with potential variable magnitude and direction of effects, and target genes nomination across tissues.

We re-analyzed the set of 115 CRF GWAS loci obtained from a single study of 72,301 unrelated UK Biobank participants from White-British ancestry (Jiang et al., 2020) to establish and examine sharing of causal signals with cis-acting GTEx e/sQTLs. This CRF GWAS set is well suited for linkage disequilibrium (LD)–informed fine-mapping, a key step to determine probabilities of causality for variants across loci. As multiple signals can reside at a locus, their identification during the fine-mapping step is increasingly recognized to improve colocalization analysis (Barbeira et al., 2021; Hukku et al., 2021; Wallace, 2021). The CRF loci analyzed here overlap 36 reported keratoconus risk loci, two of which are also Fuchs' endothelial corneal dystrophy (FECD) risk loci, and an additional FECD risk locus, *TCF4*.

Our analysis capitalizes on the latest GTEx release (v8) providing both eQTL and sQTL data and methodologies taking into account locus allelic heterogeneity (Barbeira et al., 2021). Thus, we considerably expand prior investigations which leveraged GTEx, v7, data. One used PrediXcan (Gamazon et al., 2015) to nominate CRF GWAS causal genes based on significant correlations between trait value and genetically predicted gene expression level in skin fibroblast–derived cells, deemed the most relevant (Simcoe et al., 2020). The genetic variants utilized in this type of approach are, however, not necessarily causal for the GWAS of interest, increasing the number of correlated non-causal associations (Wainberg et al., 2019). Another prior investigation used colocalization method which assumed only one causal signal per locus and restricted search to CRF signals with a highly likely causal variant (Jiang et al., 2020). Here, we also further utilize recent release of transcriptome at single-cell resolution for human cornea (Collin et al., 2021) to evaluate nominated candidate genes’ expressions in the relevant tissue.

We reasoned that integration of e/sQTLs detected in GTEx tissues, despite the relevant target tissue not being included, could deliver a subset of plausible causal gene and variant candidates for altering corneal resistance. Given accessibility of cornea tissue, these might provide tractable targets for postnatal therapeutic interventions.

## 2 Results

### 2.1 Colocalization of CRF GWAS loci with GTEx v8 cis-eQTLs and -sQTLs

Fine-mapping of the analyzed 115 CRF GWAS loci using DAP-G (Wen et al., 2016), to match the method deployed to narrow-down causal variants underpinning eQTLs and sQTLs signals in 49 GTEx tissues (Barbeira et al., 2021), yielded 181 95% credible sets (CS) of causal variants (Supplementary Table S1), with about a third (32%) of the loci harboring multiple signals. These CS matched closely, albeit not perfectly, those obtained previously (Jiang et al., 2020) using FINEMAP (Benner et al., 2016), a different Bayesian method (Supplementary Figure S1). No 95% CS are defined here for CRF locus 11 (closest gene to lead variant *EFEMP1*) and locus 100 (closest gene *ALDH3A1*), the latter also a keratoconus GWAS locus.

Five missense variants with CS posterior inclusion probability (PIP) greater than 99%, located in *ABCA6*, *ADAMST17*, *FBN2*, *GLT8D2*, and *WNT10A*, have previously been discussed (Jiang et al., 2020). Six missense variants with PIP ranging from 0.55% to 22.3% might underlie other associations, based on Combined Annotation Dependent Depletion scores (Kircher et al., 2014) greater than 20, indicating functional impact, at the same (*GLT8D2*, p.M273V) or other (in *COL6A2*, *ITIH3*, *PTPN13*, *WDR31*, and *ZHX3*) loci. The vast majority of the 5,177 candidate causal variants (Supplementary Table S1) is non-coding following variant effect predictor (VEP) (McLaren et al., 2016) annotations: 62% intronic, 15% upstream or downstream genes, and 3% intergenic.

**FIGURE 1:**
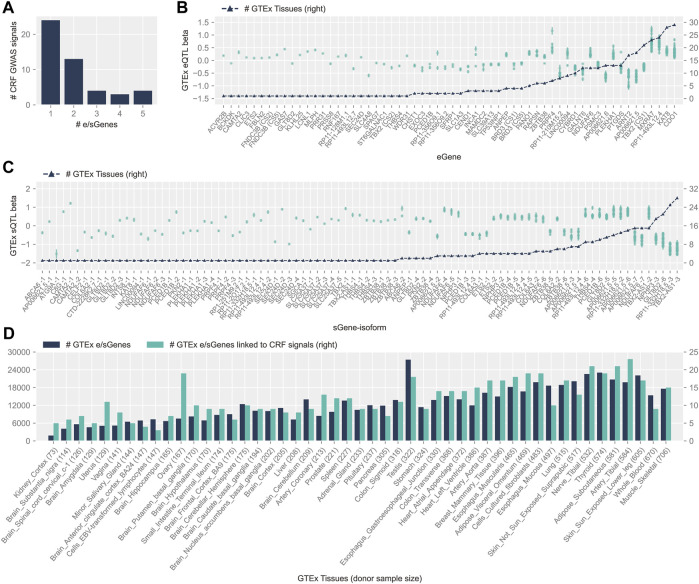
Features of colocalizing GTEx v8 cis-e/sQTLs and CRF GWAS signals. **(A)** Distribution of the number of e/sGenes nominated at CRF GWAS signals. **(B)** GTEx cis-eQTL effects (betas), ordered by increasing number of tissues where colocalization with CRF signals was detected (black triangles and scale displayed on right *y*-axis). Effect on mRNA (left *y*-axis scale) is reported for the CRF increasing allele of the lead CRF variant, with standard error of estimate displayed by a bar. Independent signals linked to the same eGene are indicated by unique credible set ID shown in parentheses. **(C)** Same as **(B)** for cis-sQTLs; target sGenes on the *x*-axis are followed by isoform ID (isoform information details can be found in Supplementary Table S3). **(D)** Plots of the number of e/sGenes linked to cis-e/sQTLs from the whole of the GTEx repertoire (dark color) and from the subset colocalizing with CRF GWAS signals (light color), across tissues. The latter are sorted by increasing donor sample size shown in parentheses.

Ninety-nine (55%) of the 181 CRF CS overlapping those defined for cis-e/sQTLs in GTEx tissues were subjected to colocalization analysis (Supplementary Tables S2, S3). The colocalization support obtained from two methods—fastENLOC regional colocalization probability (RCP) and colocalization posterior probability (CLPP)—was highly correlated, with Pearson’s R of 0.92 (*p*-value 3.52 × 10^−157^) and 0.87 (*p*-value 6.83 × 10^−96^) for cis-eQTL and cis-sQTL, respectively (Supplementary Figure S2). Forty-eight (26.5%) CRF signals colocalized with GTEx cis-e/sQTL signals, implicating 73 genes (Supplementary Figure S3), with most often one but up to five genes nominated per signal (Figure 1A). Hence, 38 (52%) candidate causal genes are not the nearest gene to the lead variant (with the highest PIP), which include 18 that are not the nearest genes to any variant in CRF CS (*AP006621.6*, *CEND1*, and *PANO1*; *ATG9A*; *C8G*, *CLIC3*, *LCNL1*, and *PTGDS*; RP1-251M9.2; *INTS8*; *PCED1B*; *RP11-128M1.1*; *RP11-210M15.2*; *SLC1A3*; *SLC39A13*; *SLC4A8*; *ST6GALNAC1*; *RP11-332H18.5*).

Loci with allelic heterogeneity where independent CS pointed to the same unique target gene strengthen causal gene candidacy. At both CRF loci 81 and 105, along with the CS composed of a highly likely (PIP > 99%) predicted functional coding variant, at respectively *GLT8D2* (pTyr24Cys) and *ABCA6* (p.Cys1359Arg), other sets colocalize with eQTL and sQTL (for *GLT8D2*) or with an sQTL (for *ABCA6*). Two other loci have two CS each co-localizing with eQTLs for the same unique eGene: *FNDC3B* (in different tissues, adipose–visceral omentum, and tibial nerve) and *TBX2* (with effect detected solely in muscle–skeletal tissue for one of the two signals, and the other detected in 20 additional tissues). We have previously highlighted *GLT8D2* and *ABCA6* relevance to cornea biology (Jiang et al., 2020). The former encodes a glycosyltransferase, substrates of which might comprise components of the proteoglycan and glycoprotein–rich extracellular matrix (ECM) of the cornea, and the latter is a top upregulated gene in cultured corneal fibroblasts from granular cornea dystrophy patients (Choi et al., 2010). Fibronectin type III domain containing 3B has an endoplasmic reticulum (ER) membrane location and been implicated in ER and secretory homeostasis (Fucci et al., 2020); zebrafish deficient in its paralog Fndc3a display severe ECM alterations (Liedtke et al., 2019). TBX2 encodes for a T-Box transcription factor 2 that has been implicated in a syndromal cardiovascular and skeletal disorder (Liu et al., 2018)**.**

About half of the colocalizations (58% with eGenes and 56% with sGenes) were found in more than one tissue. For those, the direction of effect on molecular traits for the lead variants’ CRF increasing allele was consistent across tissues for all sQTLs and 30 out of 35 (86%) eQTLs (Figures 1B,C). The direction of effects that these variants might have on gene expression in the cornea can thus be advanced with stronger support. Concordant with cis-e/sQTLs detection in GTEx (Aguet et al., 2020), the number of genes linked to colocalizing signals in each tissue was donors’ sample size dependent, with a Spearman’s correlation of 0.75 (*p*-value 4.7 × 10^−10^). Considering this bias, the cell type heterogeneity within tissues, and the overall small number of colocalizing signals per tissue, the apparent increased sharing of CRF signals with e/sQTLs in the uterus and ovary, and in other diverse tissues such as the heart, blood vessels, and adipose tissues (Figure 1D), can only be tentatively advanced. Of note, the proportion of unique causal signals at the CRF loci colocalizing with GTEx e/sQTLs was greater, although not significant (Fisher exact test *p*-value = 0.26), for loci reportedly associated with keratoconus (55.6%) than for those loci not associated (37%).

We further utilized the published transposase-accessible chromatin data for two immortalized cell lines derived from human cornea keratocytes (hTK) and cornea epithelial cells (hTCEpi) (Jiang et al., 2020). Most of the e/sGenes implicated as CRF target genes by colocalization (57/73, 78%) have at least one associated candidate causal variant lying in an open chromatin region (OCR) in hTK, mostly, or in hTCEpi cells (Supplementary Figure S3). This supports functional potential in the cornea, particularly in stromal cells, for those causal variant candidates affecting transcription in non-corneal tissues.

### 2.2 Bioinformatics support for variant causality at CRF loci colocalizing with GTEx sQTLs

**FIGURE 2:**
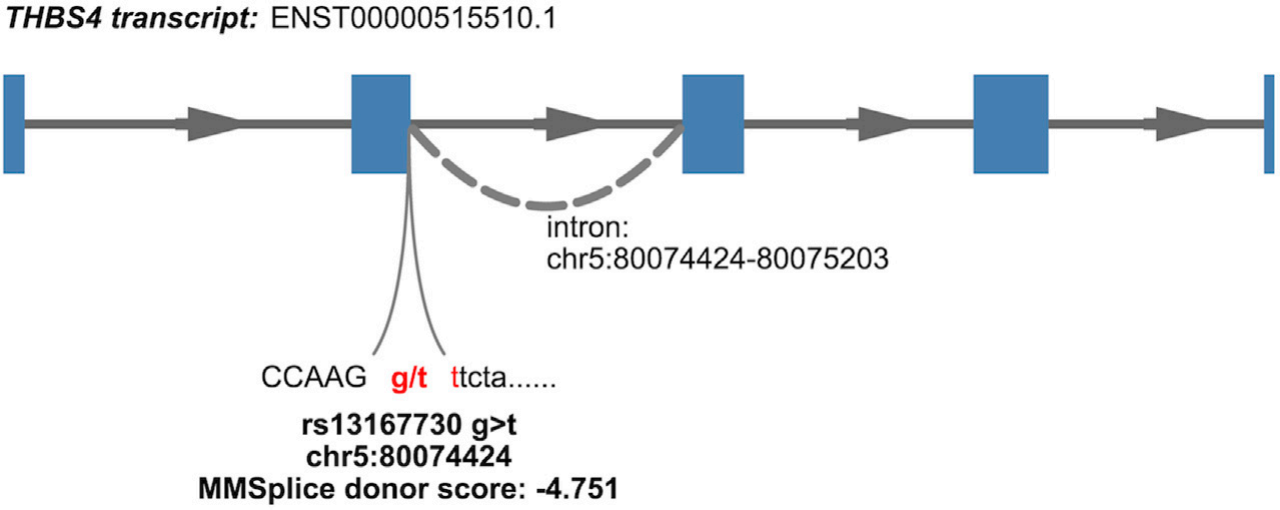
Nominated causal variant and mechanism for association at a CRF GWAS signal colocalizing with a THBS4 sQTL (isoform 4, Supplementary Table S3) detected in GTEx v8 tissues. The variant lies within a canonical donor splice site, dinucleotide gt, indicated in red; annotation of isoform is from Ensembl release 108 and GRCh38 coordinates.

Some variants in the CRF CS can be prioritized by our results and genomics input. The lead variant (PIP = 0.538) of the single signal at CRF locus 39, rs13167730, a VEP-annotated splice donor variant, is highly likely to be causal. It is located at the very 5′-end of the differentially excised *THBS4* intron in a splicing event implicated in nine tissues (Figure 2; Table 1), with the CRF increasing G allele associated with increased excision event and more favorable splice donor site [G to T transition −4.751 MMSplice donor score (Cheng et al., 2019)]. No other shared sQTL and CRF causal variant candidate localizes at a canonical intronic dinucleotide acceptor or donor site flanking an implicated splicing event. As previously noted within GTEx data (Aguet et al., 2020), splice donor or acceptor variants represent only a small fraction of sVariants, in line with diverse sequences influencing splicing outside core dinucleotide splice sites (Wang and Burge, 2008). Functional genomic predictions in these intronic and exonic regions are still challenging and driven by machine learning methods such as MMSplice (Cheng et al., 2019). The reference (ref) alleles at five of six putative CRF causal variants localized within 25 bp of a splice junction site highlighted by colocalization with GTEx sQTL show concordant predicted (MMSplice) and observed splicing effect directions (Table 1), supporting causality and mechanism.

**TABLE 1:** CRF credible set variants locating within 25 bp of splice junctions implicated by colocalization of CRF signals with GTEx sQTLs. Variant ID indicates variant chromosome:position in GRCh38 coordinates:reference allele:alternate allele. MMSplice score indicates the predicted change in effect on splicing for the alternate when compared to the reference allele. PIP is the variant posterior inclusion probability to CRF 95% credible set of causal variants.

Variant ID (rsID)	Splicing-increasing allele		PIP (CRF increasing allele)	sGene	Intron excised	Reference transcript	GTEx tissues
	Predicted (MMSplice score)	GTEx					
chr1:88805891:T:C (rs786906)	C (acceptor 0.787)	T	0.1 (T)	*PKN2*	88805672–88805890	ENST00000370521.7 (Exon 11 -> Exon 12)	Pancreas,
						ENST00000370513.9 (Exon 10 -> Exon 11)	skin Sun exposed lower leg
chr4:118816730:T:C (rs3775839)	T (donor intron −0.076)	T	0.05 (C)	*SEC24D*	118815727–118816743	ENST00000506622.5 (Exon6 -> Exon5)	Testis
chr5:80074424:G:T (rs13167730)	G (donor −4.75)	G	0.54 (G)	*THBS4*	80074424–80075203	ENST00000515510.1 (Exon2 -> Exon3)	Adipose subcutaneous, adipose visceral omentum, breast mammary tissue, esophagus gastroesophageal junction, heart left ventricle, muscle skeletal, pituitary, testis, thyroid
chr12:47249150:T:A (rs855157)	A (exon 0.326)	A	0.02 (A)	*PCED1B*	47248302–47249134	Unnamed	Artery tibial, colon sigmoid, artery aorta, artery coronary
				*RP11-493L12.4*			
chr12:47257447:C:T (rs855175)	T (acceptor 0.142)	T	0.03 (T)	*PCED1B*	47249429–47257461*	Unnamed	Colon sigmoid, lung, artery tibial, breast mammary tissue, adrenal gland, adipose subcutaneous, small intestine terminal ileum, ovary*, artery coronary, spleen
				*RP11-493L12.4*			
chr12:104021466:C:T (rs11553764)	T (exon 0.163)	T	0.28 (T)	*GLT8D2*	104021492–104029695	Unnamed	Testis
					104021492–104049894	ENST00000360814.8 (Exon2 -> Exon1)	Artery tibial, pituitary
						ENST00000547583.1 (Exon2 -> Exon1)	
chr17:61399054:T:C (rs1476781)	T (exon −0.08)	T	0.16 (C)	*TBX2-AS1*	61393748–61399036	ENST00000590421.1 (Exon2 -> Exon1)	Nerve tibial, pituitary, prostate,
				*RP11-332H18.5*			adipose subcutaneous, ovary, stomach, spleen, uterus, thyroid, heart left ventricle**

*colocalizing sQTL in the ovary was associated with additional intron, chr12:47249232_47257461, splicing; ** colocalization with the same sQTL was detected in 21 additional GTEx tissues (Supplementary Table S3).

Causality for rs786906, located 1 bp away from a canonical acceptor site, is less conclusive. The MMSplice_acceptor score, 0.787, predicts splicing of the *PKN2* intron ending at the acceptor site to be favored by the alternate C allele, but this does not match the allelic effect on the corresponding events observed in GTEx tissues. The frequent effects of sQTLs on more than one splicing event and via mechanisms other than altering splice sites (Garrido-Martin et al., 2021) make functional prioritization of candidate causal variants at this and at the majority of loci colocalizing with sQTLs, distant from implicated splice sites, challenging.

### 2.3 Colocalization analysis of CRF GWAS loci with keratoconus GWAS loci

Summary statistics for the largest keratoconus GWAS to date (Hardcastle et al., 2021) are from the meta-analysis of multi-ethnic cohorts (36 reported risk loci, ∼89% European, 4,669 cases, and 116,547 controls), a situation that might compromise causal signals' fine-mapping and colocalization owing to the potential mixed pattern of LD around causal variants or uneven missing variants across cohorts. Additionally, the keratoconus study size is relatively small, which could prevent good definition of multiple signals at a locus. Nevertheless, surmising that the comparisons of signals obtained in independent CRF and keratoconus GWAS could provide valuable information, we explored here evidence of causal variants sharing at 18 keratoconus GWAS loci, by applying three combinations of different statistical fine-mapping and colocalization methods (Supplementary Tables S4–S7) adopted by the research community.

Overall, 17 shared GWAS causal signals (within 15 loci) were detected by at least one method, seven of which by all three methods (Table 2). Of note, half of the paired association signals do not pass the criterion we applied with the GTEx data to insure credible colocalization (Table 2), that the intersecting CS variants retain at least 50% PIP of each GWAS signal; sparsity of keratoconus GWAS meta-analysis summary statistics at these loci makes failing it likely.

**TABLE 2:** CRF and keratoconus GWAS signals colocalizing following at least one of the three methodologies deployed. More information about the CRF locus can be found in Supplementary Table S1 and description of the three combined fine-mapping and colocalization methods, DAP-G–fastENLOC, SuSiE–COLOC, and FINEMAP–CLPP, in the Materials and Methods section, with full results detailed in Supplementary Tables S4–S7. cs id: credible set identifier. Same signal: whether the colocalizing CRF signals found by different methods are likely identical (sum of posterior inclusion probabilities for shared variants across credible sets is larger than 50%); eGenes/sGenes: the genes implicated by GTEx v8 e/sQTL and CRF colocalizing DAP-G–defined signals; no QTL: no GTEx v8 credible sets overlapping with signals; no Coloc: at least one variant has available GTEx v8 QTL information but no significant colocalization was found; NA: no e/sGenes implicated as those were determined using DAP-G–defined credible sets; in red: sum of PIP for variants shared with CRF/keratoconus cs is lower than 50%.

	DAP-G–fastENLOC		SuSiE–COLOC		FINEMAP–CLPP		Same signal	Nearest gene (nearest coding gene)	eGenes	sGene
CRF locus	CRF cs id	Kerato cs id	CRF cs id	Kerato cs id	CRF cs id	Kerato cs id				
4	1	1	1	1	1	1	TRUE	*LINC00970* (*ATP1B1*)	no QTL	no QTL
6	1	1	1	1	\	\	TRUE	*C1orf132 (CD34)*	no Coloc	no Coloc
29	3	3	3	5	\	\	TRUE	*TMEM212*	no QTL	no QTL
	2	2	2	2	1	2	TRUE	*TMEM212*	*FNDC3B*	no QTL
36	2	1	1	1	\	\	FALSE	*RP11-94D20.1* (*MOCS2*)	no QTL	no QTL
60	4	1	\	\	\	\	\	*RP11-473E2.4* (*COL5A1*)	no QTL	no QTL
61	1	1	1	1	1	1	TRUE	*LCN12*	PTGDS, PRR31, LCNL1, CLIC3	C8G
69	1	1	1	1	1	1	TRUE	*CMB9-55F22.1* (*PDDC1*)	*CEND1*, *PANO1*, *AP006621.5*, *AP006621.6*	*AP006621.5*
77	1	1	1	1	1	1	TRUE	*GALTN6*	*SLC4A8*, *GALNT6*	no QTL
83	1	1	1	1	\	\	TRUE	*FOXO1*	no Coloc	no QTL
90	1	1	1	1	1	1	TRUE	*SMAD3*	no QTL	no QTL
	2	2	\	\	\	\	\	*IQCH*	no Coloc	no Coloc
98	1	1	1	1	1	1	TRUE	*CAMTA2*	*CAMTA2*, *RNF167*, *SPAG7*, *INCA1*	*CAMTA2*
100	\	\	\	\	1	1	\	*ALDH3A1*	NA	NA
103	1	1	1	1	\	\	TRUE	*SGCA*	no Coloc	*SGCA*
112	1	1	1	1	\	\	TRUE	*STK35*	*RP11-128M1.1*	no QTL
115	\	\	\	\	1	1	\	*COL6A2*	no Coloc	*COL6A2*

For five of the “consensus” shared signals, the causal genes could be nominated from colocalization of CRF and GTEx QTLs (Table 2). At the two other “consensus” shared causal signals, near *LINC00970* and *SMAD3*, respectively, at CRF locus 4, also a FECD locus, and locus 90, the very stable fine-mapped CS across methods and independent GWAS (Supplementary Table S4) strengthen causal candidacy of non-coding variants: lead variant rs1200108, with PIP ranging from 0.40 to 0.69, for the former and three variants (rs12913547, rs12912010, and rs12912045) for the latter. The genome-wide enhancer to target map created by activity-by-contact (ABC) model in 131 cell types and tissues (Nasser et al., 2021) does not help in linking rs1200108 to the target gene but the CS variants at locus 90 locate in an enhancer linked to *SMAD3*, based on the ABCmax score (0.033) in transformed *MCF10A* human mammary epithelial cell line.

Overall, the two fine-mapping methods SuSiE and DAP-G return very similar CS, and five colocalizations agreed by both fastENLOC and COLOC, implicating the sGene *SGCA* and the eGene *RP11-128M1.1*.

Colocalizations only detected by DAP-G/fastENLOC (with the closest coding genes *COL5A1* and *IQCH*) appear poorly supported when examining CS overlap, in contrast to those only detected with FINEMAP/CLPP at two loci. At the first locus, the variant with the highest PIP in the solely FINEMAP-defined signal (rs4646785, intronic *ALDH3A1*, *PIP* = *0.26*) forms the keratoconus credible set defined by all three fine-mapping methods (PIP >0.95). This most supported causal variant falls in an enhancer linked to *ALDH3A1* by the ABC method (ABCmax = 0.15 in PC-9 cells, derived from human lung carcinoma). This is a very plausible candidate gene encoding for a crystallin protein with an important UV protection role, among others, in the cornea (Estey et al., 2007). At the other locus, the CRF credible set is nearly identical using DAP-G, SuSiE, or FINEMAP (one of four CS at this locus) and colocalized with *COL6A2* sQTL in GTEx tissue.

Colocalization with keratoconus loci was not detected for three CRF loci (54, 56, and 97), with the nearest protein coding genes *NDUFAF6*, *MPDZ*, and *ZNF469*; these loci display well-correlated association patterns (Supplementary Figure S4). The lead SNPs in these three signals are in high LD with many other variants and the association signals strong, making the ranking of the lead variant plausibly highly sensitive to sampling variation.

We also note other undetected but likely shared causal variants at two additional loci. The reported lead keratoconus GWAS variant (rs142493024, *p*-value = 9 × 10^−12^) at locus 35 (Hardcastle et al., 2021) is a low-frequency variant missing from summary statistics, along with the other intronic *COL6A1* variants forming one of four CS delineated at CRF locus 115 by DAP-G (cs1), SuSiE (cs1), and FINEMAP (cs4) (Supplementary Table S4). One of the multiple signals at CRF locus 29 has similarly no paired signal in keratoconus data with unique credible variant rs7635832 (PIP >0.97 by all three fine-mapping methods) missing; shared causal signal is strongly supported by variant rs4894414 in high LD (r^2^ = 0.88) with this missing variant forming a keratoconus credible set (PIP>0.95 all fine-mapping methods). The CRF fine-mapped causal variant was not linked to GTEx e/sGene and neither variant at this locus located within enhancers of published ABC catalog (Nasser et al., 2021).

### 2.4 Main target corneal cell types for CRF GWAS loci

**FIGURE 3:**
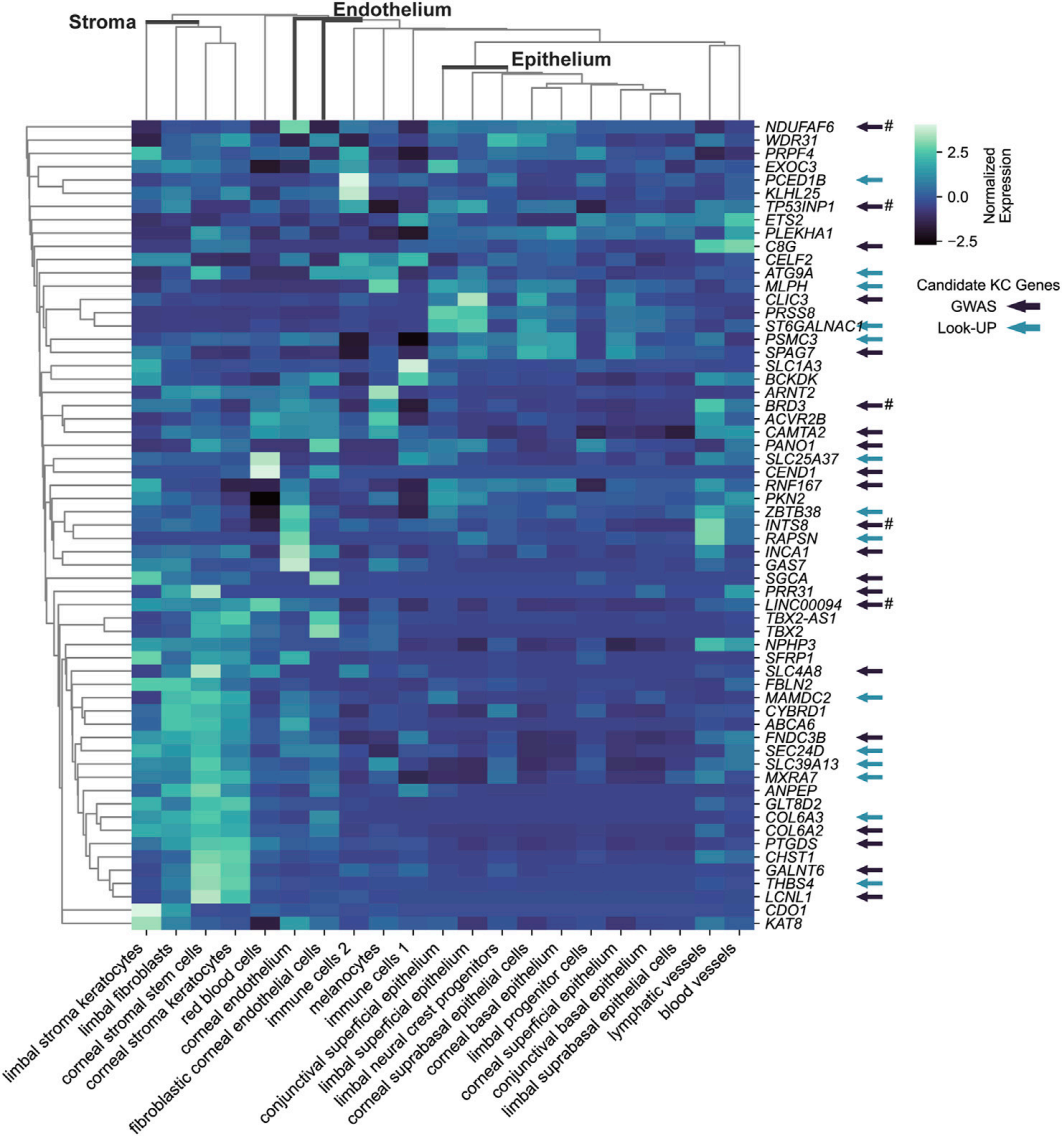
Transcriptional expression level for CRF candidate target e/sGenes across cell types of the human adult cornea cell atlas. Subset of keratoconus candidate causal genes is indicated by arrow: in dark colors, those implicated by keratoconus GWAS; in light colors, those implicated by associations using CRF or CCT associated variants (look up). # indicates that there is no evidence that the CRF and keratoconus overlapping GWAS signals are the same from colocalization analysis. Unsupervised clustering based on normalized expression levels from Collin et al (2021) analysis was performed using the *clustermap* function in python package *seaborn* v0.12.2.

**FIGURE 4:**
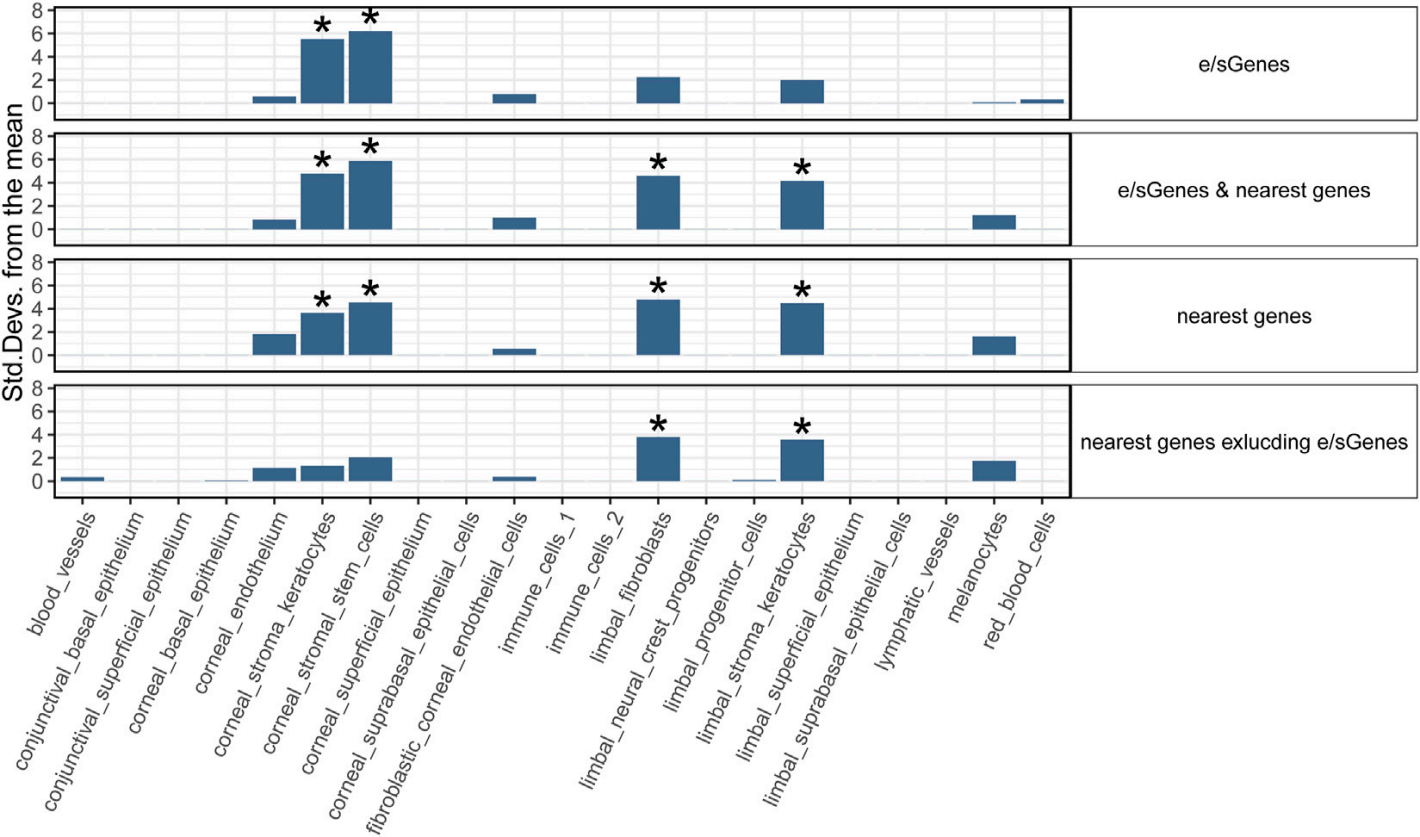
Cell type enrichment of CRF candidate target genes in human adult cornea. Four target gene sets are tested: 1) e/sGenes: e/sGenes nominated by colocalization of CRF and GTEx v8 QTL signals, 2) e/sGenes & nearest genes: e/sGenes and the nearest genes to lead variants of CRF fine-mapped signals, if not already e/sGenes, 3) nearest genes, and 4) nearest genes excluding nominated e/sGenes. *y*-axis: the number of standard deviations from the mean expression level was found to be in the target gene set, relative to the mean expression level from bootstrap-generated gene sets, sampled without replacement from data set gene list. *: significant enrichment (Benjamini–Hochberg corrected *q*-value ≤ 0.05).

To strengthen support for genes implicated by colocalization with GTEx e/sQTLs participating in corneal phenotypes and provide context for their function, we examined their transcript level in corneal and pericorneal cell types of adult human cornea using a recently released single-cell atlas (Collin et al., 2021). All 59 implicated protein-coding genes and two long non-coding RNA *TBX2-AS1* and LINC00094 (alias BRD3OS) have normalized expression levels equal to or above 1 in at least one cell type (Figure 3). Unsupervised hierarchical clustering based on these levels led to biologically meaningful grouping of cells with separation of stromal, endothelial, and epithelial cells suggesting cell type informative expression (Figure 3). The two corneal stroma cell types, keratocytes and stem cells, clustered tightly together, with high-level co-expression (normalized expression level ≥ 2) of six eGenes or sGenes: *LCNL1*, *CHST1*, *GALNT6*, *THBS4*, *PTGDS*, and *GLT8D2*. The latter three were also marker genes (logFC ≥ 0.25, compared to all remaining cell clusters) for these two cell types (Collin et al., 2021). Two limbal cell types, fibroblasts and stroma keratocytes, were further partitioned together with corneal stroma cells in a stromal cluster showing co-expression (normalized expression ≥ 1) of *COL6A2*, *COL6A3*, and *FNDC3B*. Significant enrichment for candidate CRF e/sGenes expression was found in corneal stromal stem cells and keratocytes among the tested cell types (Figure 4). Considering that GTEx-nominated e/sGenes represent candidate target genes for only a subset, 26.5%, of CRF signals (48 out of 181 GWAS signals), we also performed enrichment analysis using this gene set augmented with the nearest genes for all 181 CRF GWAS signals, and with the nearest genes only (Figure 4). The heuristic “nearest genes to lead SNPs” method for nominating causal genes has been shown on an exemplar data set to have high recall value but lacking in precision (Nasser et al., 2021). Enrichment with the nearest genes was significant in all four stromal cluster cell types, corneal and limbal, with that in corneal cells clearly driven by e/sGenes. Of note, no epithelial cell type shows enrichment whichever gene set was tested, while some enrichment in endothelial cells is detected but not strong enough to reach significance. The stronger gene sets enrichment in corneal stromal cells when e/sGenes complement the nearest genes list and the contrasted difference in enrichment magnitude between corneal and limbal keratocytes for the nearest genes (small) and e/sGenes (large) (Figure 4) suggest selection bias in causal genes nomination from regulatory signals detected in GTEx tissues.

Conditional enrichment analysis was performed to investigate whether the significant enrichment in a given cell type is independent of that in other cell types (Supplementary Figure S5). For CRF-identified e/sGenes, no significant enrichment remains when either corneal stroma keratocytes or stem cells are conditioned on, indicating that enrichment is largely driven by a shared set of expressed genes. A hint of genes specifically enriched in the contributing stem cells is notable but enrichment does not reach significance. For the same gene set with the nearest genes added, the enrichments detected in corneal stroma keratocytes and stem cells show similar dependency, in line with these enrichments being driven by identified e/sGenes. Conditional analyses also confirmed limbal stromal keratocytes and fibroblast enrichments detected with this gene set are driven mostly by genes distinct from those driving corneal stromal cell types’ enrichment. Of note, the two limbal cell types enriched do not show complete dependency and a significant enrichment in limbal fibroblast remains after conditioning on limbal stromal keratocytes expression.

The top 10% ranked genes based on cell-specificity metrics for each individual cell type are listed in Supplementary Table S8.

## 3 Discussion

Using the GTEx data, a resource combining dense genotyping data with molecular traits uniformly acquired across tissues collected post-mortem (Aguet et al., 2017), we obtained from colocalizing CRF and cis e/s-QTL signals both gene products that could participate in phenotypic outcome and the nature, and potentially the identity, of causative variants. This approach to nominate plausible causal genes, despite their absence in GTEx of the target tissue in which they most likely mediate the effect on phenotype, was vindicated by experimental follow-up in a recent investigation of bone mineral density associations (Al-Barghouthi et al., 2022). Here, its potential value is supported by several lines of evidence.

The analysis of cornea single-cell transcriptomic profiles showed that the expression of the identified CRF target genes is enriched in cells from the corneal stroma. This is in line with the abundant highly organized collagenous ECM produced by these cells, being a major determinant of the biomechanical properties of the cornea (Yang et al., 2022). Indeed, 13 of the 73 GTEx-nominated genes encode for core matrisome constituents (*COL6A2*, *COL6A3*, and *FBLN2*), glycosylation and sulfation enzymes that are likely to affect proteoglycans and glycoproteins–rich ECM (keratan sulfotransferase *CHST1*, *ST6GALNAC1*, *GLT8D2*, and *GALNT6*), an endoplasmic reticulum to Golgi export component (*SEC24D*) involved in procollagen trafficking (Lu et al., 2022), a component of the sarcoglycan complex anchoring cells to the ECM (*SGCA*), and ECM remodeling actors and regulators (*LINC0094*, *MXRA7*, *THBS4*, and *SFRP1*) (Subramanian and Schilling, 2014; Wang et al., 2020; Piipponen et al., 2022; Shen et al., 2023). An additional eGene, the zinc transporter encoding gene *SLC39A13*, is mutated in spondylodysplastic Ehlers–Danlos syndrome (OMIM 612350), a rare syndrome with multi-tissues manifestations: skeletal dysplasia, blue sclera, muscular hypotonia, and ocular impairments that include myopia and keratoconus. The ECM plays essential structural and physiological roles in all tissues, in health and disease (Frantz et al., 2010) and tissue sharing of gene products impacting on its, tissue-diverse, composition indicated by a wide range of systemic manifestations in inherited monogenic connective and musculoskeletal tissue disorders (Callewaert et al., 2008; Voermans et al., 2008).

Our results leveraging genetic control of gene expression in tissues other than the cornea suggest some level of regulatory instructions sharing for ECM-concerned genes across tissues. The gene encoding sarcoglycan A is an interesting target gene considering that the closest coding gene at an intergenic unresolved signal in CRF locus 82 encodes for another subunit of the sarcoglycan complex, SGCG, and both SGCA and SGCG are known to bind, in the muscles, to the ECM component biglycan (Rafii et al., 2006) that is encoded by the closest gene to a strong CRF signal reported on the X chromosome (Simcoe et al., 2020), not analyzed here. Biglycan also interacts with type VI collagen (Wiberg et al., 2002), strongly causally implicated in CRF determination by our present analysis and that of rare coding variants (van Hout et al., 2020) and both implicated in regulating ECM stiffness and maximal load (Leiphart et al., 2021). The anchoring of cells to the ECM via bridging interactions is thought to play a critical role in mechanosensing and signaling, processes that have not been previously highlighted in the context of CRF or keratoconus GWAS interpretations but recognized important players in cornea biology (Yang et al., 2022), and potential drivers of keratoconus pathology (Dou et al., 2022). Among its multiple roles, *THBS4* has been shown to influence trafficking of sarcoglycans in myocytes (Brody et al., 2018), but the function of the specific isoform modulated by causal splicing variant highlighted by our study remains to be determined. *PLEKH1*, also known as *TAPP1*, encoded by an implicated eGene, is one plausible effector of signals transduced by sarcoglycans’ associated macromolecular complexes as recruited by syntrophins, adapter proteins, in fibroblasts (Hogan et al., 2004); it also interacts with cytoplasmic tyrosine phosphatase *PTPN13* (Kimber et al., 2003), implicated by a credible set coding variant (pE1630K).

The fraction of all CRF-associated causal variant and gene candidates exposed here are likely relevant to more than one tissue and involved in homeostatic rather than (or as well as) developmental processes. These are important considerations for the identification of potential therapeutic targets for postnatal interventions. Many CRF GWAS variants show associations with other traits and diseases and significant genetic correlations reported with ocular and, at lower strength, non-ocular traits such as blood pressure and respiratory capacity (Simcoe et al., 2020). Colocalization would enable transfer of knowledge and hypothesis on causal mechanisms across these traits and diseases. Combined genomic and fine-mapping evidence have well established that the majority of GWAS signals reside in putative enhancer regions, but the relative lack of their colocalization with detected eQTL, even when target tissues are surveyed, has been much remarked upon (Connally et al., 2022). Assuming false negative results from colocalization and power are minor or non-issues, the most obvious explanation is that the right cellular context is not interrogated or masked in bulk RNA analysis of heterogeneous tissues. The genes causally implicated by colocalizing CRF and GTEx e/sQTLs appear biologically relevant, but our enrichment analyses show them to be skewed toward those expressed in two corneal stromal cell types when compared to the, larger, set of genes nearest to signals. These show significant enrichments in two additional, limbal, cell types, which indicates an inherent bias in the representation of cell types (or states) in the GTEx data resource and/or plausible higher specificity of genetic regulation of implicated genes expressed in the limbal cell types. The current and future growing focus on cataloging molecular traits’ associations in specific cellular contexts should help unmask some missed regulatory links between variants and genes (van der Wijst et al., 2020; Balliu et al., 2021; Neavin et al., 2021).

It has been argued that eQTL mapping efforts might, however, never fully evidence all missing cis regulatory GWAS hits as GWAS and eQTL detections operate under different premises (Mostafavi et al., 2022). For example, variants with small effect on the transcript level of genes that are tightly regulated and critical during development have little power to be detected by eQTL analysis, yet are likely to have a strong effect on the phenotype. This might be the case for the candidate coding genes previously highlighted as strongly supported by their role in Mendelian cornea and/or connective tissue disorders (Iglesias et al., 2018; He et al., 2022) which are notably absent from the e/sGenes; they are *UBIAD1* (CRF locus 1 cs1) implicated in Schnyder corneal dystrophy (OMIM 121800), *DCN* (CRF locus 79), *TGFB2* (CRF locus 7 cs1), *SMAD3* (CRF locus 90), *COL5A1* (locus 60 cs2, cs3, and cs4), *ZNF469* (CRF locus 97 cs1) implicated in stromal cornea dystrophy (OMIM 610048), Loeys–Dietz syndrome types 4 (OMIM 614816) and 3 (OMIM 613795), and classical Ehlers–Danlos (OMIM 130000) and Brittle cornea (OMIM 229200) syndromes, and all are potential keratoconus susceptibility genes. All, but *TGFB2*, are the closest coding genes to lead variants (highest PIP in CS), and for all, the lead variants are located 13–590 kb [median 92.5 kb] away from their promoter, in putative enhancer regions. One of these enhancers affecting *SMAD3* is the most supported in transformed cells subjected to tamoxifen treatment (Ji et al., 2018) among a wide range of cells surveyed using the ABC method (Nasser et al., 2021), supporting that some of the enhancers and associated variants might be revealed only under specific challenges. In the adult corneal and pericorneal tissues' single-cell transcriptomics data examined, *ZNF469* and *COL5A1* showed high specificity of expression (>60%) in the limbal stroma keratocytes, a cell type (or state) not significantly enriched in target genes implicated by colocalization with GTEx e/sQTLs. The non-detection of highly likely target genes as eGenes could thus be due to non-representation in GTEx of cell types/states in which variants exert their effects. It remains to be seen whether or not they will be detected in future eQTL efforts or by other means, and how specific in time, space, and environment their regulatory function might be.

Other limitations of our and similar studies lie in the means to ascertain that causal variants are shared, i.e., colocalization of fine-mapped signals, as both statistical fine-mapping and colocalization have limitations (Hukku et al., 2021; Wallace, 2021). Regions with multiple causal signals that are physically close and in some level of LD, which fine-mapping algorithms might resolve differently, requirements for LD reference matching summary statistics and consequences of insufficiently powered studies or missing data for the proper enumeration of CS to be compared, the choice of prior probability of colocalization for Bayesian methods are all influencing parameters. Methods to identify errors or heterogeneity in GWAS summary statistic (Chen et al., 2021) could have been deployed to remove problematic markers or loci but missing data would remain a major problem. Increasing availability of whole genome sequences for large data sets on which well-powered GWAS can be performed, such as that of a quantitative measure like CRF in the UK Biobank, will make those GWAS the best suited for colocalization analyses.

We noted some variations in the eQTL colocalization results between our current and previous analyses of the subset of CRF loci; while the analysis should have improved by taking into account the locus heterogeneity in both data sets and GTEx analysis being more powered in v8 than in v7, the more stringent criteria that we applied to ensure credible colocalization and differences in methodology all contributed to this variation and missed potentially true shared signals. *GLT8D1*, for example, is biologically supported by another glycosyltransferase *GLT8D2* being implicated at another locus by two independent causal signals. The CRF signal passed thresholds for colocalization with *GLT8D1* eQTL in both methods applied in our current study but failed the additional criterion introduced.

In conclusion, despite pitfalls and restricted search space, the insight gained from integrating CRF GWAS with cis e/sQTL from non-corneal tissues represented in GTEx v8 provided many functional pointers, for 26.5% of CRF signals, guiding prioritization for experimental validation. The subset of CRF associations highlighted is biased but therapeutically interesting as genetic effects detected in GTEx and expression of nominated genes in the adult cornea suggest entry points into homeostatic, rather than the less targetable developmental, processes.

## 4 Materials and methods

### 4.1 Colocalization of CRF GWAS signals with GTEx v8 cis e/s-QTLs

Readily available fine-mapping results for cis-e/sQTLs signals for all v8 GTEx tissues were downloaded from the public repository at https://zenodo.org/record/3517189#.Y-0UwcfP2Ul.

Fine-mapping of causal signals at CRF GWAS loci was identically performed, using DAP-G (Wen et al., 2016). LD information was calculated by plink v1.90b4 (Chang et al., 2015) option *--r* using the CRF GWAS sample of 72, 301 unrelated UK Biobank participants of White-British ancestry (Jiang et al., 2020). The 95% CS were constructed using the script get_credible_set.pl (https://github.com/xqwen/dap/tree/master/utility) following DAP-G run with parameter *-msize* set to 5. Colocalization of causal signals was performed using fastENLOC v2.0 (Wen et al., 2017) with default parameters, which returns both a single variant colocalization probability (SCP) and a regional colocalization probability. The required annotation files (VCF format) for GTEx v8 e/sQTL DAP-G results were prepared using an in-house python script (https://github.com/xinyixinyijiang/CRF_GTExv8_KC) combining information from available {tissue}.variants_pip.txt.gz and {tissue}.clusters.txt.gz files (https://zenodo.org/record/3517189#.Y-0UwcfP2Ul). CRF GWAS variants' IDs were formatted to match the GTEx v8 variant ID (format: chromosome_position_ref_alt_build) in build GRCh38, using pyliftover v0.4 (https://github.com/konstantint/pyliftover), lifting coordinates from the genome build GRCh37 to GRCh38. We used GTEx_Analysis_v8_sQTL_groups.tar.gz from the GTEx data portal (https://gtexportal.org/home/datasets) for mapping the sQTL introns to the corresponding genes. The signal colocalization posterior probability (CLPP) (Hormozdiari et al., 2016) was calculated using the DAP-G fine-mapping results with the formula described in Gay et al. (2020):

$$CLPP=1-\prod_{i=1}^{K}\left(1-{PIP}_{GWAS,i}\times {PIP}_{QTL,i}\right),$$

where K is the number of common variants between GWAS and QTL overlapping CS, and PIP is the posterior inclusion probability of variants to each credible set of causal variants.

The thresholds for colocalization were set to 0.01 and 0.1 for CLPP and fastENLOC RCP, respectively, following Gay et al. (2020) and Barbeira et al. (2021), and we used the union of these methods to declare colocalization. Higher thresholds of 0.1 and 0.5, respectively, for CLPP and fastENLOC indicate strongly supported colocalizations. After visual inspections of the colocalizing signals using LocusCompare (Liu et al., 2019), the two quality criteria were added to filter out dubious colocalization results: i) GTEx cis-e/sQTLs with the false discovery rate (FDR) larger than 5% and ii) CRF-e/sQTLs paired signals not encompassing the important contributing variants to original signals in their intersect (those for which the sum of PIPs for overlapping variants was lower than 0.5 in either study).

### 4.2 Colocalization with keratoconus GWAS

With no standard or exact way to conduct statistical fine-mapping and colocalization, we deployed the most currently adopted algorithms and practices, all of which account for potential multiple independent GWAS signals within a single genomic region.

Three Bayesian fine-mapping methods, which differ in the priors used, and in the approach taken to compute posterior inclusion probabilities: DAP-G (Wen et al., 2016), SuSiE (Zou et al., 2022), and FINEMAP (Benner et al., 2016) were paired, respectively, with fastENLOC v2.0 (Wen et al., 2017), COLOC v5.1.0 (Wallace, 2021), and CLPP (Gay et al., 2020). The keratoconus GWAS summary statistics was downloaded from Supplementary Data 15 of Hardcastle et al. (2021). LD information was derived from the CRF GWAS sample. Of note, DAP-G and SuSiE do not always return a credible set of causal variants, while FINEMAP does.

For fastEnLOC, the CRF GWAS DAP-G fine-mapping results (Section 4.1) were used. The same DAP-G pipeline was used to generate keratoconus GWAS fine-mapping results, which were summarized into VCF format by the script summarize_dap2enloc.pl (https://github.com/xqwen/fastenloc/tree/master/src) and provided to fastENLOC using the -eqtl command. FastENLOC was executed with the default settings.

For SuSiE and FINEMAP fine-mapping, performed for both CRF and keratoconus GWAS, LD information was calculated using the LDSTORE v2.0 (Benner et al., 2017) with default parameters. The COLOC package was run with the functions *runsusie* (for fine-mapping) and *coloc.susie* (for colocalization) with all parameters set to default. For CLPP, we used the CRF fine-mapping results obtained with FINEMAP, previously published (Jiang et al., 2020). The keratoconus GWAS fine-mapping results for CLPP were generated using FINEMAP with option *–sss* and the variant priors extracted from PolyFun (approach 1 implemented in the function extract_snpvar.py), which uses the precomputed prior causal probabilities based on 15 UK Biobank traits meta-analysis (Weissbrod et al., 2020).

### 4.3 Nearest gene identification

The nearest gene of each CRF fine-mapped variant is identified using the function “closest-features” in the software BEDOPS v2.4.41 (Neph et al., 2012), with the argument “--closest --dist”. The same gene annotation for GTEx v8, GENCODE v26, was downloaded from the GTEx data portal, transferred to bed file format (function gtf2bed), sorted (function sort-bed), and used for finding the nearest gene or protein-coding gene.

### 4.4 Human adult cornea cell type enrichment

The human adult cornea singe-cell expression matrix based on RNA-seq and metadata were downloaded from http://retinalstemcellresearch.co.uk/CorneaCellAtlas (i.e., with cell type annotations kept as per the authors’ analysis (Collin et al., 2021)). These data were input into the R package expression weighted cell type enrichment (EWCE) v1.5.7 as a cell type data set (CTD) (Skene and Grant, 2016). When creating the CTD data, this software calculates for each gene a cell type specificity metric that represents the proportion of the average transcript level in cells of a particular cell type relative to the average across all cells. This measure is thus independent of the expression level, and genes with low expression that might appear highly cell specific are removed (Skene and Grant, 2016). The EWCE function bootstrap_enrichment_test was used for calculating enrichment metrics, using 10,000 repetitions, and the options genelistSpecies and sctSpecies were set to “human”. The “controlledCT” option in bootstrap_enrichment_test was used for conditional analysis. To generate reproducible results, the random seed for the bootstrap was set to 1.

## Data Availability

The data sets used in this study can be found in online repositories. The names of the repositories can be found in Material and Methods section and in CRF and Keratoconus GWAS publications (Jiang et al., 2020; Hardcastle et al., 2021). The codes used for this study can be found in the GitHub repository https://github.com/xinyixinyijiang/CRF_GTExv8_KC. The data sets generated are fully presented in Supplementary Material.
