# Supplementary material for: Colocalization of corneal resistance factor GWAS loci with GTEx e/sQTLs highlights plausible candidate causal genes for keratoconus postnatal corneal stroma weakening
Source: Front Genet. 2023 Aug 9;14:1171217. doi: 10.3389/fgene.2023.1171217 (PMC10445647; doi:10.3389/fgene.2023.1171217)
Supplement: Supplementary file 1 [file DataSheet1.PDF]

Supplementary Figures

Supplementary Figure 1: Comparison between FINEMAP and DAP-G fine-mapping results for 115 CRF GWAS loci.
(A) Number of credible sets per CRF locus defined by FINEMAP and DAP-G
(B) The posterior inclusion probability (PIP) values for variants in credible sets defined by either FINEMAP or DAP-G.
(C) The number of CRF loci (labelled) harbouring 0 to 5 credible sets defined by DAP-G and FINEMAP.

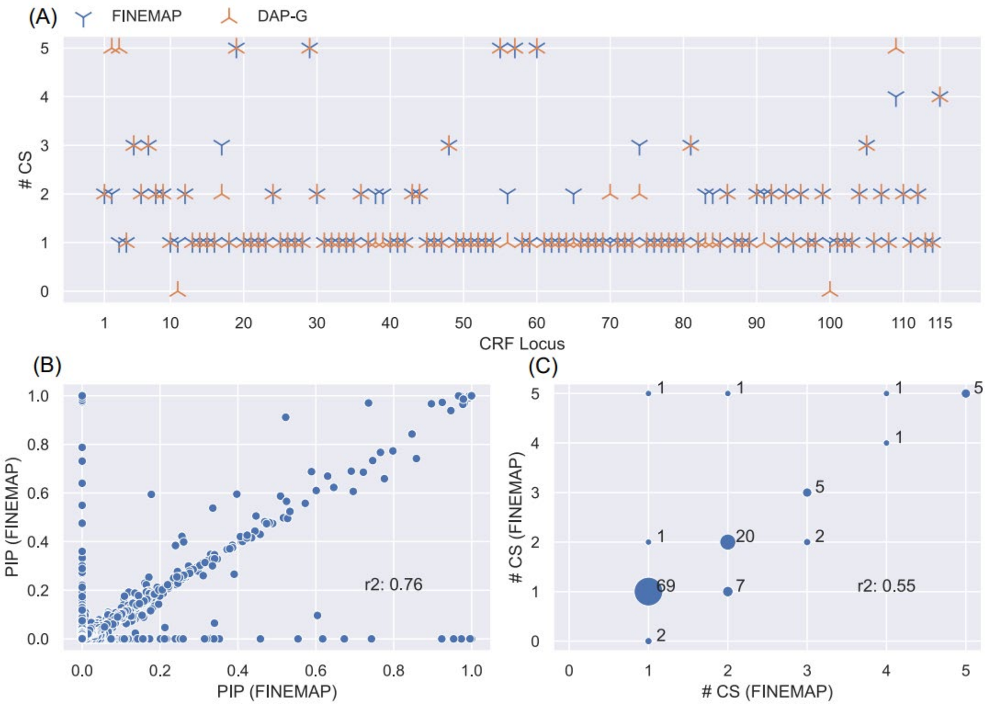

Supplementary Figure 2: RCP and log10(CLPP) values for overlapping CRF and GTEx cis-eQTL (left) and cis-sQTL (right) signals that passed quality criteria (e/sQTL with FDR <= 5% and sum of PIPs for overlapping variants higher than 0.5 in both studies).

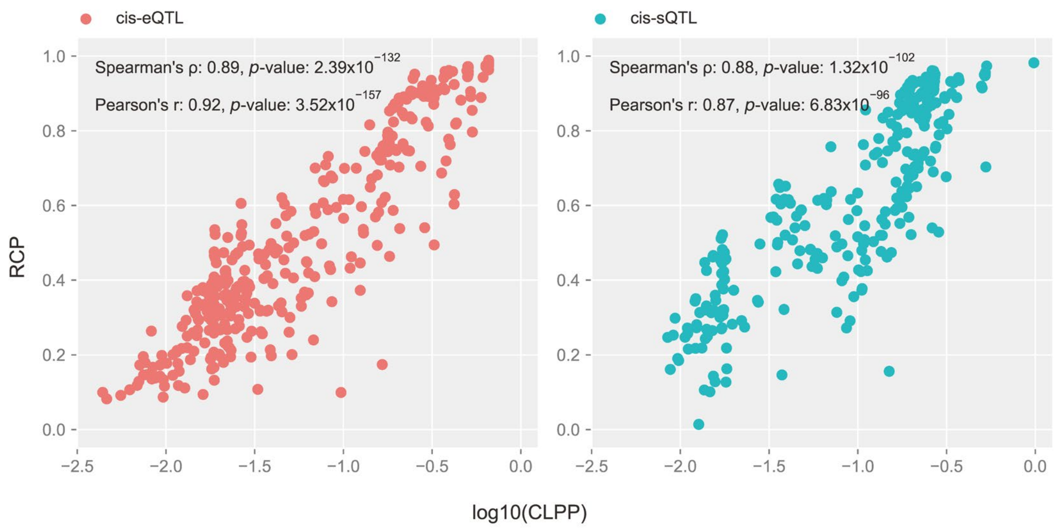

Supplementary Figure 3: Colocalizing GTEx v8 cis-e/sQTLs and CRF GWAS signals. The left Y-axis lists the modulated genes followed by the type of transcriptional effect (e for expression level alteration, s for splicing event alteration) and the colocalizing CRF GWAS signal (credible set) id in locus listed on the right Y-axis. Concordance of the direction of effects for lead variants in overlapping credible sets and colocalization significance are indicated respectively by orientation and size of the colocalization symbol (triangle). Inverted triangles denote a sQTL affecting multiple isoforms in one tissue with opposite direction of effects on splicing events. Color coding of triangles indicates signals overlapping open chromatin regions (OCR) in hTK and/or hTCEpi corneal cell lines or not.

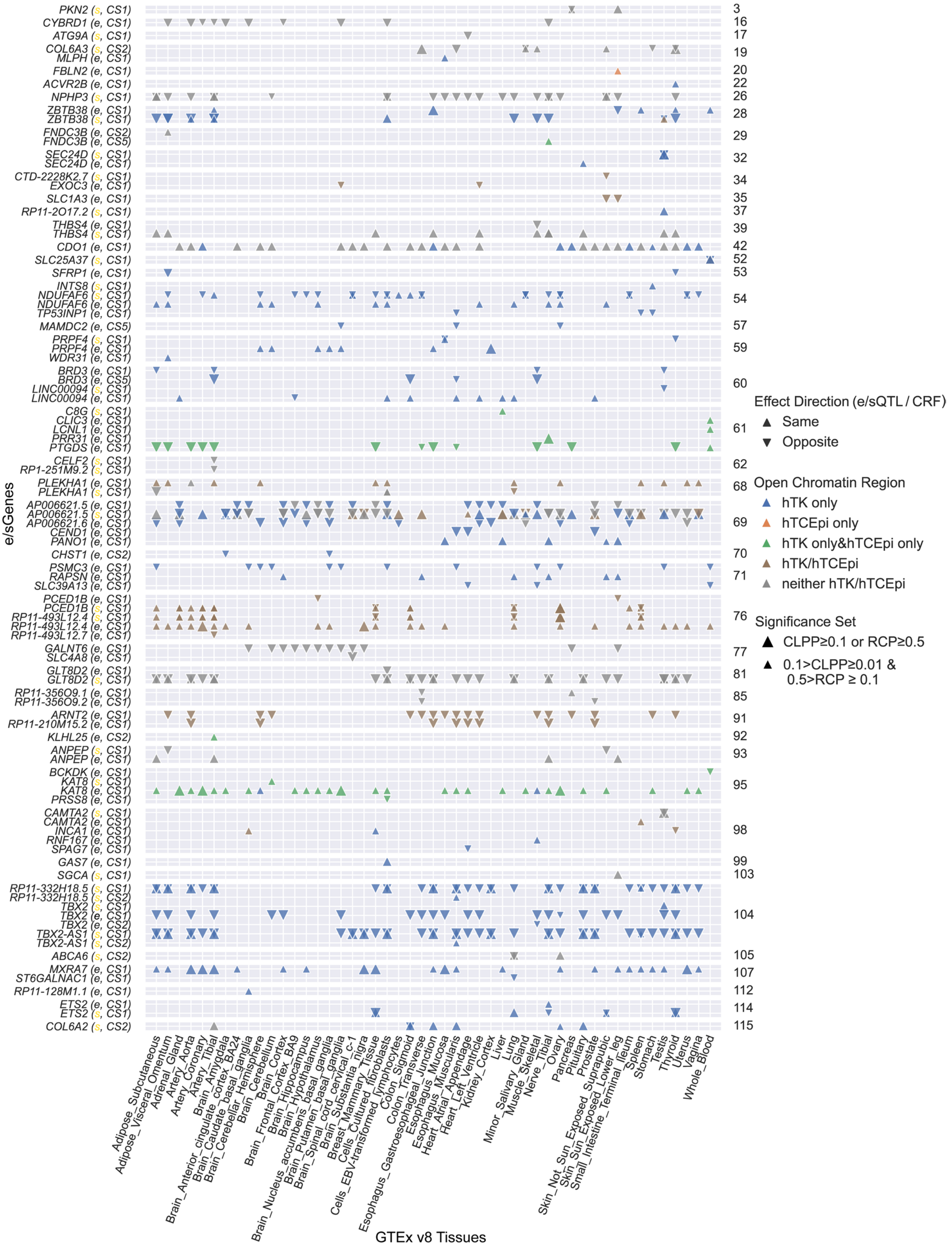

Supplementary Figure 4: Comparisons of GWAS p values from three CRF-keratoconus overlapping loci: CRF loci 54, 56 and 97.

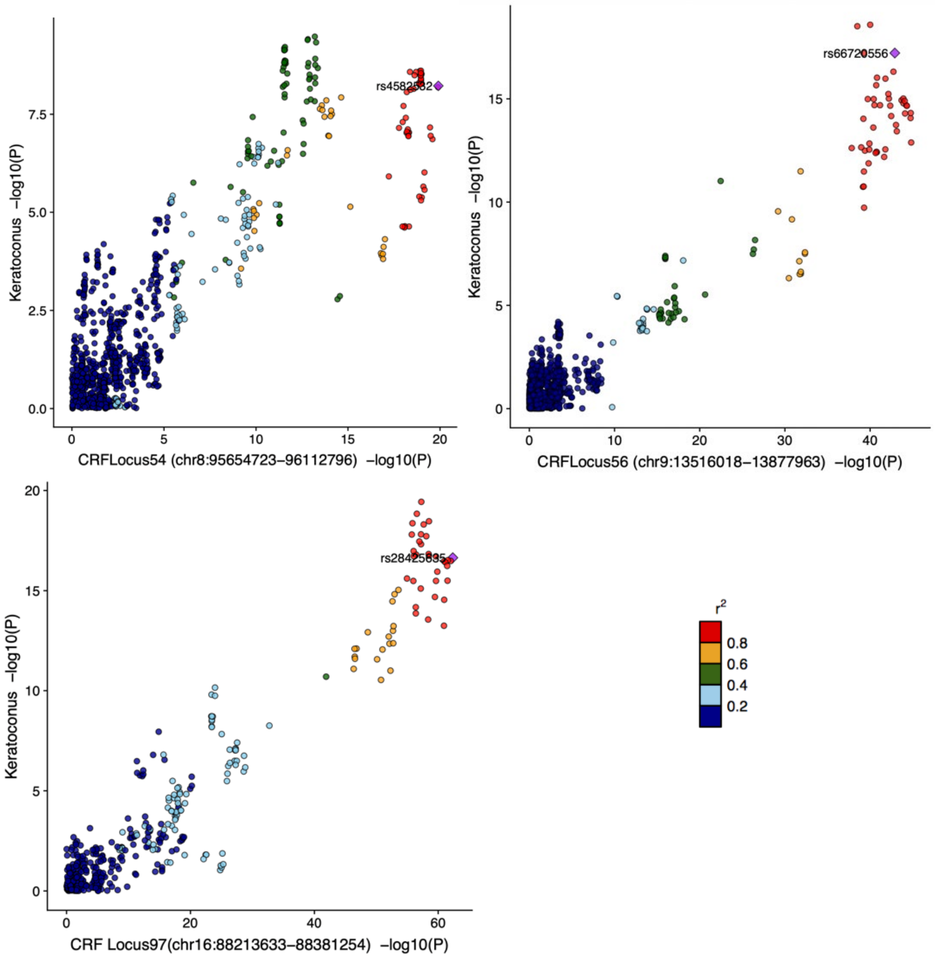

Method: The LocusCompare(Liu et al., 2019) online version was used for plotting and selecting the top variant (highlighted in purple), which is defined by having the lowest sum of p-values from both studies by default. Colors shown are the linkage disequilibrium (r^2, from 1000 Genomes Phase 3(Auton et al., 2015) European population) value between variants and the variant highlighted.

Supplementary Figure 5: Conditional cell-type enrichment tests in human adult cornea cell types using 1) e/sGenes: CRF-GTEx colocalizing e/sGenes; 2) nearest genes: the nearest genes of all CRF independent signals; 3) e/sGenes & nearest genes: e/sGenes and the nearest genes of CRF signals without colocalizing e/sGenes. (*: significant enrichment, $q \leq 0.05$)

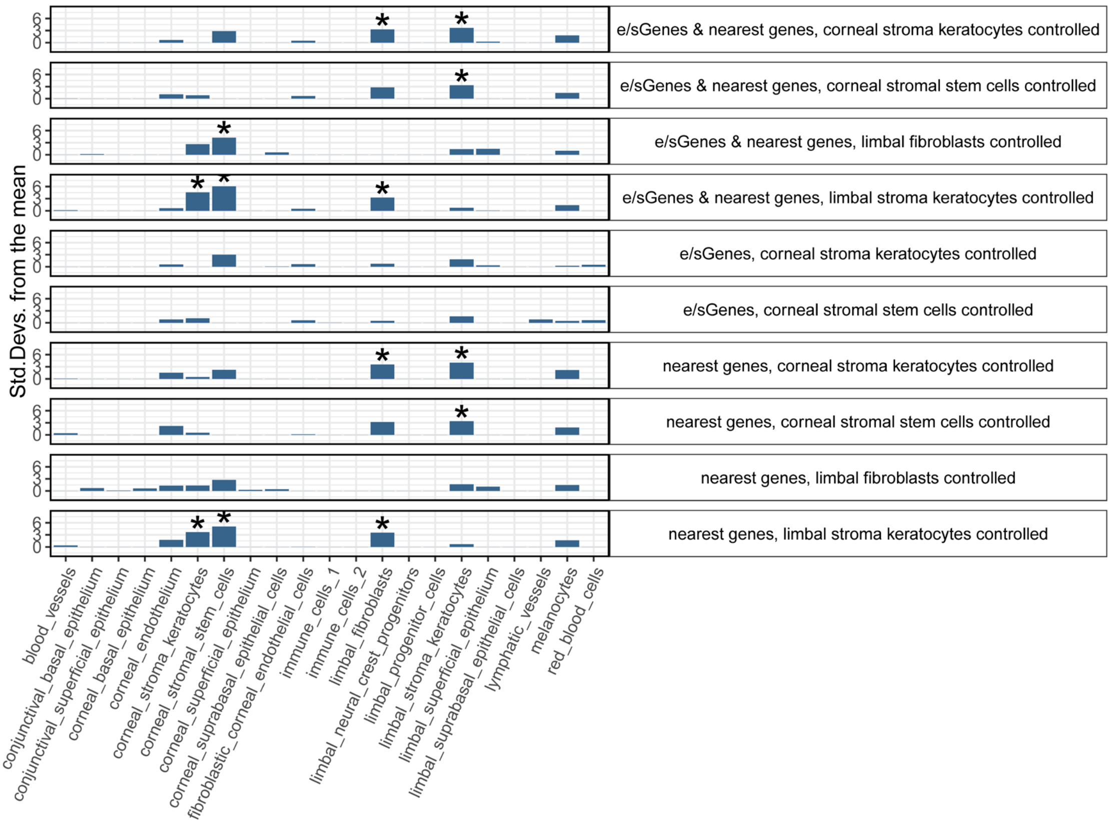

References:

Auton, A., Abecasis, G. R., Altshuler, D. M., Durbin, R. M., Bentley, D. R., Chakravarti, A., et al. (2015). A global reference for human genetic variation. *Nature 2015 526:7571* 526, 68–74. doi: 10.1038/nature15393.

Liu, B., Gloudemans, M. J., Rao, A. S., Ingelsson, E., and Montgomery, S. B. (2019). Abundant associations with gene expression complicate GWAS follow-up. *Nature Genetics 2019 51:5* 51, 768–769. doi: 10.1038/s41588-019-0404-0.
